# Survival Analysis Part IV: Further concepts and methods in survival analysis

**DOI:** 10.1038/sj.bjc.6601117

**Published:** 2003-08-26

**Authors:** T G Clark, M J Bradburn, S B Love, D G Altman

**Affiliations:** 1 Cancer Research UK/NHS Centre for Statistics in Medicine, Institute of Health Sciences, Old Road, Oxford OX3 7LF, UK

**Keywords:** survival analysis, missing data, validation, repeated events

## INTRODUCTION

In the previous papers in this series (Bradburn *et al*, 2003a, 2003b; Clark *et al*, 2003), we discussed methods for analysing survival time data, both univariate and multivariate. We have dealt with only a portion of the methods available for analysing survival time data, and in many cases, useful alternatives to (or extensions of) these methods exist. We have also left unanswered other questions regarding the design and analysis of studies that measure survival time and, in particular, dealing with situations where some standard modelling assumptions do not hold. We conclude this series by tackling these issues. These ideas are described in a question and answer format, and introductory references are provided for the reader to investigate further.

## IN A SURVIVAL ANALYSIS, CONTINUOUS VARIABLES ARE SOMETIMES CATEGORISED.SHOULD WE DO THIS (AND IF SO, HOW)?

In medical research, it is common to see continuous measures grouped into categories to simplify a covariate's relationship with survival and its interpretation. There is no statistical reason for grouping and it can lead to as many problems as it seeks to avoid. The categorisation of a continuous covariate by definition discards data and can be seen as introducing measurement error. It also leads to biased estimates and a reduced ability to detect real relationships (Schmoor and Schumacher, 1997; Altman, 1998). Nevertheless, there are sometimes good reasons to categorise a continuous covariate in the analysis of survival (and indeed any) data. When doing so, it is wise to note the following points:

1. Use cut-points that have been predetermined rather than testing multiple values. A common choice of boundaries is fixed centiles such as quartiles. It is preferable though to use established cut-points that have clinical meaning, and therefore provide consistent groupings between studies. Examples include dividing oestrogen receptor level at 10 fmol, and age into 5- or 10-year intervals.
2. Do not choose cut-points based on minimising *P*-values, as this method gives biased results (Altman *et al*, 1994; Altman, 1998).
3. If possible, use more than two categories to reduce the loss of infor-mation and allow some assessment of the linearity of any trend.
4. Ensure that each group contains an adequate number of individuals (and events).

**Figure 1:**
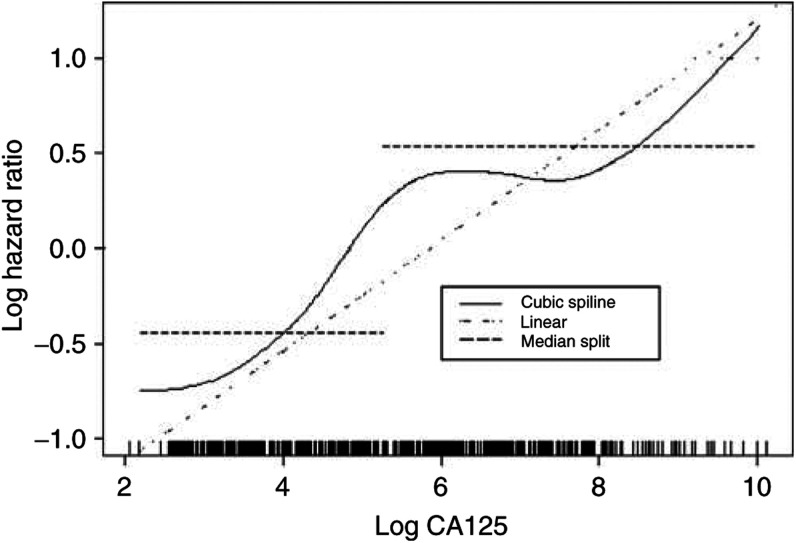
Modelling log CA125 using spline functions: ∣ corresponds to measurements.

Grouping is sometimes used because there are concerns with mismodelling the relationship when there is a nonlinear relationship between the variable and log hazard. The simplest approach is to evaluate the effect of adding a quadratic term to the model, but better approaches to use are smoothing splines (Therneau and Grambsch, 2000) or fractional polynomials (Royston *et al*, 1999). Figure 1

shows the result of modelling a new covariate, (log) CA125, in the previously used ovarian cancer data, by the method of smoothing splines (with 11 degrees of freedom). There is evidence of nonlinearity (*P*=0.002) and the plot suggests that CA125 might be modelled as a cubic effect. It is clear that modelling the data using a binary or linear variable would be inappropriate here (see Figure 1). Knorr *et al* (1992) discussed these issues in the context of prognostic studies in cancer.

## IN OUR CLINICAL TRIAL, WE COLLECTED MEASUREMENTS AT PREARRANGED VISITS. CAN WE INCLUDE MULTIPLE MEASUREMENTS FOR THE SAME COVARIATE IN OUR SURVIVALANALYSIS?

If variables measured after entry into the study are to be included in the survival model, special methods are required. Such methods are called *time-dependent* (or *updated*) *covariate methods*, as the variables they incorporate may change value over time. For example, if a longitudinal study seeks to assess the effects of smoking on cancer, a variable for each patient may be defined, being equal to 0 (nonsmoker) or 1 (smoker) at any time. If a nonsmoker begins smoking after entering the study, then this covariate is updated (from ‘0’ to ‘1’) at the time that smoking begins. This covariate contributes more information than using smoking status at time of entry alone. It is important to note that post-entry measurements cannot be validly incorporated into a survival model without using these methods.

Recall that for the proportional hazards model, the formula relating a covariate *x*_1_ to the hazard *h*(*t*) at time *t* is


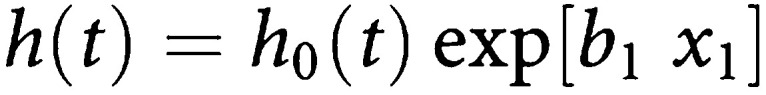


where *h*_0_(*t*) is the baseline hazard. If repeated measurements of a covariate *x*_1_ are available, the formula changes to


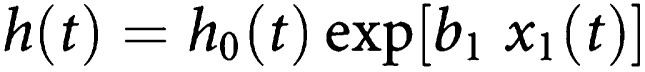


where *x*_1_(*t*) is the value of *x*_1_ at time *t.* (It is also possible to use, but harder to interpret, an accelerated failure time model here.) The covariate *x*_1_ may be continuous or categorical, and may change freely or at fixed time intervals. The coefficient *b*_1_ represents the additional relative hazard for each unit increase in *x*_1_ at any given time. This model is different from models with *time-dependent coefficients* (Bradburn *et al*, 2003b), in which the *effect* of a covariate changes rather than the value of the covariate itself, that is, *h*(*t*)=*h*_0_(*t*) exp[*b*_1_(*t*) *x*_1_].

The time dependent method can be applied in many standard statistical software packages. However, the approach described requires a large amount of data and is therefore rarely seen. One also has to be confident that the collection process is not *itself* dependent on clinical progress, perhaps by using scheduled assessments. Further details of the method, and some precautions, are noted in Altman and De Stavola (1994).

## MOST SURVIVAL ANALYSIS METHODS ASSUME THE CENSORING IS NONINFORMATIVE. WHAT IF THE CENSORING IS INFORMATIVE?

Informative censoring occurs when individuals are lost to follow-up for reasons that may relate to their (unknown) outcome. For example, in a randomised trial in which the main outcome is time to cancer recurrence, a patient who is lost to follow-up may be more likely to have experienced drug toxicity or ill health and thus may also be more susceptible to (earlier) relapse. Informative censoring introduces bias into the standard methods discussed previously. Unfortunately, it is difficult both to identify informative censoring and to assess its impact. It is helpful though to know what proportion of censored individuals were lost to follow-up before the end of the study (Clark *et al*, 2002).

A simple, *ad hoc* approach to the problem is to perform sensitivity analyses, to assess the impact of assigning different survival times to those patients whose observed (censored) survival times may have been affected in this manner. For example, if a patient suspected to be in ill health exits the study at 4 weeks, a first analysis may be performed with this patient censored at 4 weeks and a second where the patient is assumed to have relapsed at 4 weeks (i.e. a ‘best case – worst case’ scenario). This approach works best when there are few such patients, but in that situation, the possible bias will be very small. Another possibility is to decide *a priori* that all such patients will be treated in a particular way. The issue has been of particular concern in randomised trials of nicotine replacement therapy, in which losses to follow-up are considerable. In a systematic review of randomised trials, patients who were lost to follow-up were regarded as being continuing smokers (Silagy *et al*, 2002).

More formal approaches have been proposed (e.g. Robins, 1995a, 1995b; Scharfstein *et al*, 2001). In general, they assume that a relationship exists (and can be modelled) between censoring times and baseline covariates and perhaps also post-treatment patient data. It is difficult to evaluate the assumptions of these complex methods, and implementation in statistical software is limited.

If follow-up stops because the patient has experienced a different defined event, the problem may be viewed as a competing risk scenario (see below), or handled via a mixture model (or ‘cure’ model), where the differing event types are explicitly modelled. The latter method makes particular sense if the two events are quite dissimilar, such as patient recovery and patient death.

In practice, if there is little informative censoring, the bias introduced to standard methods is minimal, and in general using these along with simply reporting loss to follow-up (perhaps with a basic sensitivity analysis) will suffice. Good patient follow-up and avoidance of unnecessary drop-out is by far the best solution, and when and why drop-out occurs should always be reported (Moher *et al*, 2001).

## SOME COVARIATE DATA ARE MISSING IN OUR ANALYSIS. WHAT SHOULD WE DO?

Missing data are a common problem when developing survival models in cancer. Individuals without complete covariate data are usually omitted, but the resulting analysis has reduced power and may be an unrepresentative subset of patients. Often many covariates have missing data, and the absence of a small percentage of data points for each variable can lead to a greatly depleted sample. Unless only a few values are missing, some investigation of the missing data and methods that accommodate it should be considered. In the ovarian cancer data set presented previously, a small number of important factors containing little or no missing data were used. The database contains several other factors in which missing data were frequently encountered, and a more definitive analysis (Clark *et al*, 2001) was able to incorporate these factors, while retaining all patients by applying multiple imputation methods (Van Buuren *et al*, 1999). Multiple imputation is a framework in which missing data are imputed or replaced with a set of plausible values. Several data sets are then constructed, each being analysed separately, and their results are combined while allowing for the uncertainty introduced in the imputation. Other approaches exist (e.g. Lipsitz and Ibrahim, 1998), but imputation approaches have more software available (Horton and Lipsitz, 2001). Further details, discussion and references are given in another analysis of the ovarian data found in Clark and Altman (2002).

We recommend that authors of research papers are explicit about the amount of missing data for each variable and indicate how many patients did not have complete data. Imputation techniques are powerful tools and are increasingly available in software, but are not a panacea. Inherent in the method is the assumption that a model relating data absence to other measured covariates (and possibly survival too) exists and can be specified. This has much in common with the situation where informative censoring is suspected, and similarly, their practical experience is limited at the present time. Researchers should be aware of the assumptions, most of which are untestable, and use sensitivity analysis to assess the robustness of results. Ultimately, these problems are best avoided by minimising missing data.

## HOW SHOULD WE CHOOSE WHICH VARIABLES TO INCLUDE IN OUR SURVIVAL MODEL?

In some cases, the factors to be included in the model will be predetermined. In many others, there will be several possible covariates from which only a handful are to be chosen. This is often because there are a large number of covariates of which some are unimportant, but the identification and elimination of these is not always easy. As a starting point, it is good practice to include known prognostic factors and any that are specifically required by the study aims (e.g. the treatment identifier in the analysis of a clinical trial). It is then the burden of new factors to add significant additional predictive ability (Simon and Altman, 1994).

If there are a large number of factors of interest and there is relatively little information about their prognostic influence, automated selection techniques such as stepwise methods can be used. There are variations on these that start either with all covariates (backward elimination) or none (forward selection), adding or removing covariates according to statistical significance at some predecided level. A disadvantage of both is that they only evaluate a small number of the set of possible models. Instead, each possible model could be fitted, with the best being picked on the basis of a goodness-of-fit measure such as Mallow's C (Hosmer and Lemeshow, 1999). However, this may be time-consuming with many covariates, multiple testing is problematic, and is seldom used due to its noninclusion in many software packages.

Unfortunately, all these methods are problematic. The ‘best’ model is derived solely on statistical grounds (and indeed may lack any clinical meaning), the regression coefficients produced are biased (too large) and standard errors and *P*-values are too small, especially for smaller sample sizes and when few events occur. Backward elimination is possibly the best of the above methods for identifying the important variables, and it allows one to examine the full model, which is the only fit providing accurate standard errors and *P*-values (Harrell, 2001). An alternative, the lasso method (Harrell, 2001) attempts to force some regression coefficient estimates to be exactly zero, thus achieving variable selection while shrinking the remaining coefficients toward zero to reflect the overfitting and overestimation caused by data-based model selection.

If one cannot completely prespecify a model, it may be best to apply backward elimination or lasso to a full model of prespecified covariates of interest, and use bootstrap methods to compare the stability and predictive accuracy of the full model with that of a reduced one (see next question for further details).

## WE HAVE DEVELOPED A PROGNOSTIC MODEL FOR OVERALL SURVIVAL. HOW CAN WE MEASURE ITS PREDICTIVE ABILITY? HOW CAN THE MODEL BE VALIDATED?

In survival analysis, statistical models are employed to identify or propose combinations of risk factors that might predict patient survival. It follows that to be of use, the model must be able to: (1) make unbiased predictions, that is, give predicted probabilities that match closely those observed, and (2) distinguish higher and lower risk patients. These are the two components of predictive ability, and are called *calibration* and *discrimination,* respectively. Importantly, models rarely perform as well on either basis when used to predict survival in patients other than those used to derive the model. A model that closely mirrors the survival patterns of the present data is said to have *internal validity*, but to be of wider use should do so for other groups of patients as well (be *externally valid*). Before a model is applied routinely in clinical practice, it should have been shown to meet both criteria.

Measures of discrimination include the *c*-index and Nagelkerke's *R*^2^(*R*_N_^2^) (Harrell, 2001). The *c*-index, a generalisation of the area under the receiver operating characteristic (ROC) curve, is the probability of concordance between observed and predicted survival based on pairs of individuals, with *c*=0.5 for random predictions and *c*=1 for a perfectly discriminating model. Similarly, *R*_N_^2^=0 indicates no predictive ability and *R*_N_^2^=1 indicates perfect predictions. Calibration may be quantified using an estimate of slope shrinkage (Harrell, 2001). Each quantity may be evaluated for the data used in the modelling by randomly splitting the patients into two samples, one to derive the model and the other to validate it. The proportion of data to include in each sample is, however, arbitrary and although estimates of predictive accuracy from this approach are unbiased, they also tend to be imprecise. Bootstrapping, a method that involves analysing subsamples from a data set, or ‘leave-one out’ cross-validation may be more beneficial. For these analyses, an alternative is to estimate shrinkage factors and apply these to regression coefficients to counter overoptimism. These techniques allow evaluation on multiple data sets. Once the internal validity of a model has been established, it can be tested for its generalisability by applying the model to other patients, and using the above methods to assess the adequacy of the predictions.

A good summary of important issues can be found in Justice *et al* (1999) and Wyatt and Altman (1995), and more details on the statistical methods are given in Altman and Royston (2000). In summary, internal validation is necessary before a model is proposed, and external validation is highly recommended before it is to used in clinical practice.

## CAN WE PERFORM AN ANALYSIS WHERE THERE ARE UNMEASURED FACTORS THAT MAY AFFECT SURVIVAL TIME?

In practice, one cannot be sure that all important prognostic variables have been measured. In general, omitting variables will simply reduce the predictive ability of a model, so that patients with similar measured covariates will exhibit large variability in their survival. When a strongly prognostic variable is omitted, however, the model may be biased. In particular, the estimated treatment effect in a randomised trial may be biased if an important prognostic variable is not adjusted for, even when that variable is balanced between the treatment groups (Schmoor and Schumacher, 1997; Chastang *et al*, 1988). It is inappropriate to proceed at all if vital information such as clinical stage in breast cancer patients is unavailable.

Another form of missing covariate is when some individuals have a shared exposure that is unmeasured. For example, members of the same family will have shared dietary and other environmental exposures, so that their outcomes cannot be considered to be independent. A similar situation arises in cluster randomised trials and multicentre trials in general (Yamaguchi *et al*, 2002). Such data can also be considered as being ‘multilevel’, with variation both between and within groups. Random effects (or ‘frailty’) models can be used to allow covariate effects to vary across groups (O'Quigley and Stare, 2002). Such models are widely used in other contexts, in particular, in meta-analysis. Frailty can also be considered to apply to individuals, relating to the idea of unmeasured variables as a possible explanation for observed heterogeneity of outcome. Use of such models depends on precise knowledge of the frailty distribution, which is generally not available (Keiding *et al*, 1997).

Lack of fit of a Cox model may be better explained by other modelling approaches (O'Quigley and Stare, 2002), such as the accelerated failure time model (Keiding *et al*, 1997).

## SEVERAL PAPERS IN OUR RESEARCH AREA HAVE APPLIED (ARTIFICIAL) NEURAL NETWORKS AND REGRESSION TREES AS AN ALTERNATIVE TO THE COX MODEL. WHAT ARE THESE METHODS?

### Artificial neural networks

**Figure 2:**
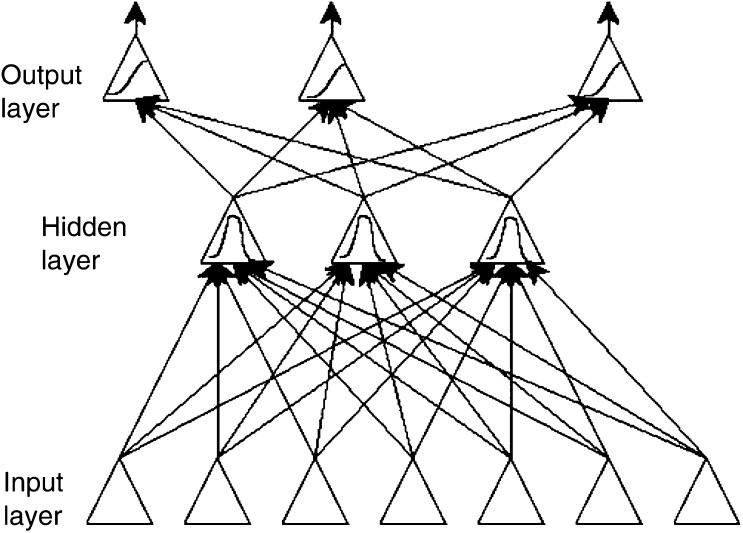
An example of an ANN.

Artificial neural networks (ANNs) are a relatively new method for assessing the extent to which a series of covariates explain patient outcomes. The key feature of the ANN methodology is to assume that there are some latent, or ‘hidden’, intermediary variables in the input (covariate) and output (survival probability) processes. The most common model is the three-layer model shown in Figure 2

. Under this model, the covariates (input) do not act directly on the response variable (output), but channel their influence into a series of latent (hidden) variables. It is the relative importance of these unobservable variables which determines the survival. For a more detailed introduction to these methods, see Cross *et al* (1995).

This methodology is appealing in that it can incorporate complex relationships between covariates and survival more easily than standard approaches such as Cox regression, which may be too simplistic. However, there have been several major criticisms of the method: (a) the high chance of overfitting the data, (b) the lack of easy interpretation of the model and of the impact of individual covariates, (c) the perceived ‘black box’ methodology involved, and (d) the difficulty in handling censored survival times. The last issue arises because it is usually the status of the individuals (i.e. alive or dead) at a given point (or points) in time that is taken to be the response. Biganzoli *et al* (1998) and others have modelled the hazard functions directly, in a promising attempt to extend this method. Reviews comparing the examples where both ANN and regression methods had been used to derive prognostic models have found that overall ANNs are little better than classical statistical modelling approaches (Sargent, 2001), and misuses of ANNs in oncology are common (Schwarzer *et al*, 2000). We therefore advise caution in their use, and the involvement of an experienced statistician.

### Classification and regression trees

**Figure 3:**
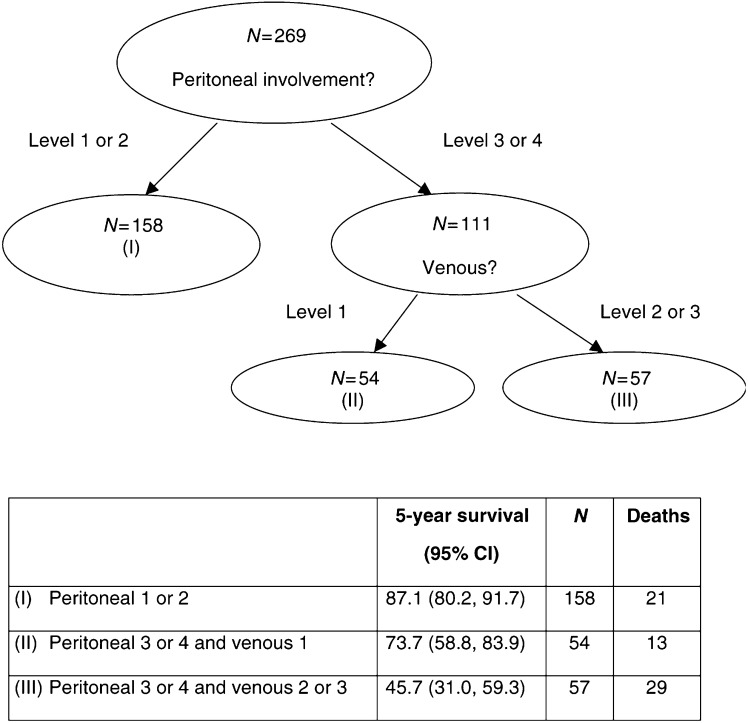
A CART for Dukes' B colonic cancer study.

The classification and regression tree (C&RT) approach is based on dividing the cohort into groups of similar response patterns, using covariates (Lausen *et al*, 1994). The partitioning algorithm starts with the covariate that best discriminates the survival outcome between two subgroups. For continuous or multicategory variables, the method thus needs to determine the threshold that best dichotomises the variable. This process is repeated for each subgroup in turn using all the available covariates. The same covariate may be used more than once, and the process stops eventually with either no covariate adequately dividing the subgroups further or when the subgroups have reached a specified minimum size. Figure 3

shows an unpublished C&RT analysis in a Dukes' B colonic cancer study, in which four categorical variables (perforation, peritoneal involvement, venous and margin) were assessed for their prognostic value in overall survival. Using a logrank test at each step, it was found that peritoneal involvement (levels 1, 2 *vs* 3, 4) discriminated best between good and bad survival, and level 1 venous subdivided patients with high levels of peritoneal involvement. The stopping rule employed was the first occurrence of either (a) the maximum logrank statistic is not statistically significant at the 1% level or (b) when any subgroup contains less than 25 patients. The latter condition ceased the partitioning algorithm in the example, yielding the three groups of patients described in Figure 3.

The major advantage of C&RT is its ease of interpretability – it reflects how many decisions are made. It also relies on fewer distributional assumptions (Schmoor *et al*, 1993) and is particularly useful in situations where there are interactions. The disadvantages of C&RT lie in having to decide what threshold to use for continuous covariates, and to correct for multiple testing and overfitting. The automated covariate selection is similar to forward stepwise methods in regression, and hence shares their problems (see the choice of covariate section). Finally, as C&RT seeks to classify patients into groups, it offers little in the way of estimated effect of risk factors. Nevertheless, C&RT is a useful complement to other methods, in particular as an exploratory tool that can inform future research.

## CAN WE ANALYSE DIFFERENT TYPES OF EVENTS OR REPEATED EVENTS?

Traditional survival analysis methods (including all those discussed so far) assume that only one type of event of interest occurs, and at most once. More advanced methods exist to allow the investigation of several types of events (e.g. cancer death, vascular death, other), or an event that may occur repeatedly (e.g. cancer recurrence). We will describe methods for each in turn.

Where the survival duration is ended by the first of several events, it is referred to as *competing risks analyses*. Analysing the time to each event separately can be misleading, and in this context the Kaplan–Meier method, in particular, tends to overestimate the proportion of subjects experiencing each event. The cumulative incidence method, in which the overall event probability at any time is the sum of the event-specific probabilities, may be used to address this. Univariate tests and statistical models also exist, and an overview of several of the methods proposed can be found in Tai *et al* (2001). Models are generally implemented by entering each patient several times – one per event type – and for each patient, the time to any event is censored on the time at which the patient experienced another event.

Where multiple events of the same type occur, it is common practice to use the first event only, but this ignores information. Three approaches to use this extra information are demonstrated using artificial patient data in Table 1

**Table 1:** Data layout under four recurrent event models with patient 1 having three events (at times 8, 12 and 26) and patient 2 having two events (at times 10, 18)

**Model**	**Patient i.d.**	**Time interval**	**Event^a^**	**Stratum^b^**
Conditional A	1	(0,8]	1	1
	1	(8,12]	1	2
	1	(12,26]	1	3
	1	(26,31]	0	4
	2	(0,10]	1	1
	2	(10,18]	1	2

Conditional B^c^	1	(0,8]	1	1
	1	(0,4]	1	2
	1	(0,14]	1	3
	1	(0,5]	0	4
	2	(0,10]	1	1
	2	(0,8]	1	2

Marginal model	1	(0,8]	1	1
	1	(0,12]	1	2
	1	(0,26]	1	3
	1	(0,31]	0	4
	2	(0,10]	1	1
	2	(0,18]	1	2
	2	(0,18]	0	3
	2	(0,18]	0	4

Independent increment	1	(0,8]	1	1
	1	(8,12]	1	1
	1	(12,26]	1	1
	1	(26,31]	0	1
	2	(0,10]	1	1
	2	(10,18]	1	1

a 1=had event of interest, 0=censored.

b Relates to the number of events and is used in the fitting of the model as the strata variable.

c Gap time model.

. In a *conditional* model, follow-up time is broken up into segments defined by events, with each patient being at risk for an *i*th event once the (*i*−1)th has occurred. Patient 1 in Table 1 is therefore assumed not to be at risk of a second event until the first has occurred, and so is at risk of experiencing this from time 8 until time 12. This model comes in two types: using either the time since the beginning of the study (type A) or the time since the previous event (type B). The *marginal* model, on the other hand, considers each event to be a separate process and, by definition, the time for each event starts at the beginning of follow-up for each patient. Here, all patients are considered to be at risk for all events, regardless of how many events they have previously had, and so patient 2, for example, was considered at risk of events 3 and 4 despite being lost to follow-up at the second. A third approach, called the *independent increment* model, is closest in spirit to a conditional model but takes no account of the number of previous events experienced by a patient, and for this reason the conditional and marginal models are often preferable. For each model, the data should be entered in the form of one patient record per event number as illustrated in Table 1.

All of the above models are usually applied within a Cox model framework, although accelerated failure time methods may equally be used. These models are fitted using the same basis as standard approaches, with two exceptions: (1) a cluster effect is used to adjust the standard errors because patients are repeated in the study, and (2) the analysis is stratified – with the exception of the independent increment method – with the event type (for competing risks) or number (for recurrent events) defining the strata. Interaction effects between covariates and strata may be used to assess whether covariate effects vary across competing outcomes or event number. For example, Kay (1986) presents an example of a treatment that reduces the risk of death from one cause, but increases the risk of death from another.

More thorough reviews of the above (and other related) methods can be found in Hosmer and Lemeshow (1999), and Therneau and Grambsch (2000). These modelling procedures are generally only a little more difficult than for single-event data, and software is widely available. As with any statistical model though, it is still important to assess its adequacy and fit. In each case, the choice of the best method of analysis will depend on the disease in question and the goals of the analysis. However, the aims such as those described here can often be highly relevant, and where this is the case these methods should be strongly considered.

## SUMMARY

Most analyses of survival data use primarily Kaplan–Meier plots, logrank tests and Cox models. We have described the rationale and interpretation of each method in previous papers of this series, but here we have sought to highlight some of their limitations. We have also suggested alternative methods that can be applied when either the data or a given model is deficient, or when more difficult or specific problems are to be addressed. For example, analysis of recurrent events can make an important contribution to the understanding of the survival process, and so investigating repeat cancer relapses may be more informative than concentrating only on the time until the first. More fundamentally, missing data are a common issue in data collection that in some cases can seriously flaw a proposed analysis. Such considerations may be highly relevant to the analysis of a data set, but are rarely mentioned in the analysis of survival data. One possible reason for this is a perceived lack of computer software, but many of the approaches discussed here are currently incorporated into existing commercial statistical packages (e.g. SAS, S-Plus, Stata) and freeware (e.g. R). On the other hand, the desire may be to ‘keep things simple for the readership’. This view is reasonable, but is valid only where a simple analysis adequately represents the survival experience of patients in the study. Ensuring the analyses are appropriate is therefore crucial. More advanced survival methods can derive more information from the collected data; their use may admittedly convey a less straightforward message, but at the same time could allow a better understanding of the survival process.

The aim of this series has been to aid awareness, understanding and interpretation of the many and varied methods that constitute the analysis of survival data. It is paramount that analyses are performed in the knowledge of the assumptions that are made therein, and the more complex methods, in particular, are best applied by a statistician.
